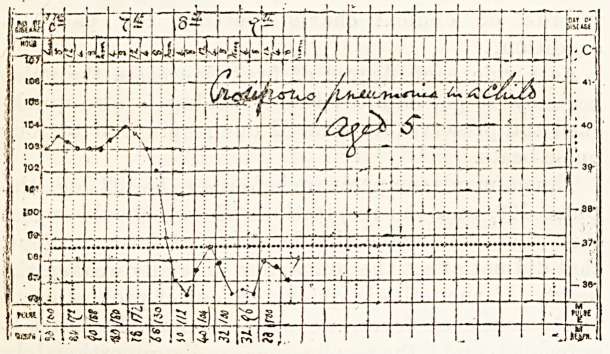# On Temperature Charts

**Published:** 1894-04-28

**Authors:** F. W. Burton-Fanning

**Affiliations:** Physician to the Norfolk and Norwich and Jenny Lind Hospitals


### Medical Progress and Hospital Clinics.

[*The Editor will be glad to receive offers of co-operation and contributions from members of the profession. All letters should be addressed to* The Editor, The Lodge, Porchester Square, London, W.]

### ON TEMPERATURE CHARTS.

By F. W. Burton-Fanning, M.B., M.R.C.P., Physician to the Norfolk and Norwich and Jenny Lind Hospitals.

So much time and labour are expended on the preparation of clinical charts that it is very desirable that the full value of these records be appreciated. The object of the following remarks is to attempt to answer the question—What can be learnt as to the nature of a patient's illness and as to his progress in it by observations of the temperature, pulse, and respirations? The majority of diseases definitely affect one or more of these three functions, and often in a way that is characteristic of the particular malady. Before considering morbid alterations of the temperature and of the frequency of the pulse and respirations, we must remember that in health they are subject to variations under certain conditions. Thus in health the temperature runs a definite course during the day, gradually rising from the morning till between five and eight p.m., when it attains its maximum, and then falling till it reaches its lowest point between two and six a.m. This tendency for morning fall and evening rise of temperature is also observed in most fevers.

The regulating mechanism is so constructed that variations in the temperature of the air, the ingestion of food, and exercise, produce no appreciable effect on the body heat, though it is lowered by excessive fatigue. The temperature of children is slightly higher than that of adults, the morning and evening fluctuations are less marked, and fever is more easily produced in them. In elderly people, on the other hand, the average maximum temperature is about a quarter of a degree lower than in persons under forty years of age.

The number of pulse beats and respirations per minute also vary at different periods of life, as shown in the subjoined table:—

| | Pulse. | Respirations. |
|---|---|---|
| At birth | 130 | 44 |
| One year | 110 | 28 |
| Four years | 100 | 25 |
| Adolescence | 72 | 18 |

It is important to observe that with variations in their frequency at the different ages the ratio of about four pulse beats to one respiration is maintained after the first year.

We have seen that the range of temperature compatible with health is a narrow one, practically speaking, between 97 degrees and 99 degrees. Though, theoretically, fever is not said to commence till 99·5 degrees is reached, experience teaches us that a temperature of 99·2 degrees is not to be ignored as an index to the possible presence of disease.

With the frequency of pulse and respirations, however, it is different, for they can be more markedly disturbed by conditions which do not amount to disease; the effects of excitement and hurry must be excluded before a quickening of pulse or respirations be recorded as symptoms of disease. Moreover, it is not very exceptional to meet with people whose normal pulse frequency may be as low as 50 or as high as 90 per minute.

Bearing these facts in mind, we must see how far the records of clinical charts portray the patient's condition.

As a rule each degree of fever increases the pulse beats by 10 per minute; the breathing is also quickened and preserves its ratio of one respiration to four pulse beats.

Thus a man's temperature is found to be 102 deg., the pulse 100, and the respirations 25; we should expect that there was some simple cause of fever present, that did not affect the lungs nor specially weaken the heart. If the pulse is quickened out of proportion to the amount of fever, we suspect that the heart is enfeebled; and if the respirations are disproportionately hurried, the lungs are probably the seat of disease.

Here is a chart which shows fever in a child of fifteen months, due to the mere irritation of cutting a tooth. For three weeks its centre for regulating the temperature was thus disordered, but recovered itself as soon as an incisor came through.

With about two degrees of fever, the pulse was raised about twenty beats above the normal for a child of that age, and the respirations were only proportionately quickened. Such slight disturbance as is involved in dentition, would only cause fever in unstable childhood, and the probability of the freedom of the heart and lungs, from inspection of the chart, assisted in the diagnosis of the cause of the child's malady. Not only is the temperature of children more readily disturbed, but the frequency of the pulse and respirations is also increased more easily and markedly in them than in adults. For this reason a single observation is of very little value in a child's illness, but the average of a series of records should be used.

No disease presents a more constantly characteristic chart than pneumonia.

The temperature, like the other symptoms, rapidly attains its full height, and is peculiarly sustained, showing but little tendency to morning intermission. In the majority of cases defervescence is also sudden, and crisis takes place more often on the seventh than any other day.

But the feature par excellence of the pneumonic chart is the marked alteration of the pulse respiration ratio, for in no other malady does the breathing become about half as quick as the pulse. In grown-up people, a pulse rate of 130 with 60 respirations per minute, indicates a severe attack, but children will often breathe 80 or 90 times a minute and have a pulse of 180, in an attack of pneumonia which ends favourably; when the pulse and respirations are quicker than this, however

I think recovery is the exception. Diminished frequency of the pulse and respirations is often the first sign of improvement, and precedes the fall of temperature. Besides illustrating most of these points, the above chart represents the subnormal temperature which so commonly follows for a few days the crisis; the heat regulator is, as it were, out of gear, and allows the temperature in falling to overshoot the mark. With this reaction of temperature there is often also depression of the heart's action, which is slow, and may be irregular.

Bronchitis contrasts with pneumonia in the less tempestuous onset of its fever, and the more gradual defervescence. The breathing is accelerated out of proportion to the pulse, but does not approach the ratio of one to two. It is remarkable in this disease that diminishing frequency of pulse is often of evil significance, as it may arise from poisoning of the brain with carbonic acid.

## Figures and Tables

**Figure f1:**
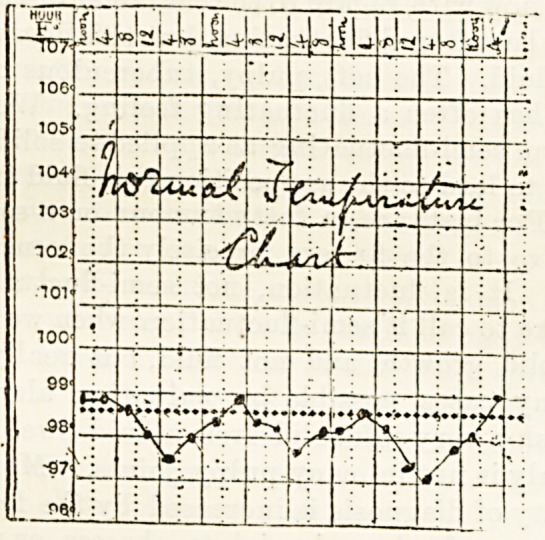


**Figure f2:**
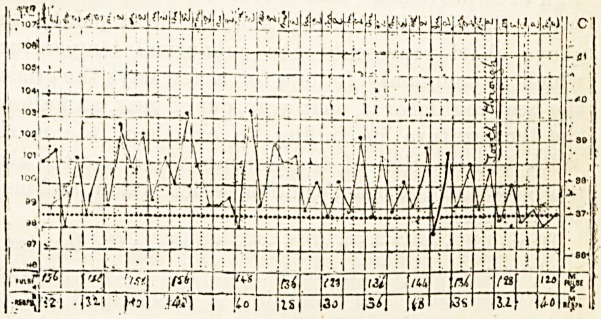


**Figure f3:**